# Verification of genes differentially expressed in neuroblastoma tumours: a study of potential tumour suppressor genes

**DOI:** 10.1186/1755-8794-2-53

**Published:** 2009-08-17

**Authors:** Kaisa Thorell, Annika Bergman, Helena Carén, Staffan Nilsson, Per Kogner, Tommy Martinsson, Frida Abel

**Affiliations:** 1 Department of Clinical Genetics, Gothenburg University, S-405 30 Gothenburg, Sweden; 2 Department of Mathematical Statistics, Chalmers University of Technology, S-412 96 Gothenburg, Sweden; 3 Department of Pathology, Lundberg Laboratory for Cancer Research, SU/Sahlgrenska, S-413 45, Sweden; 4 Childhood Cancer Research Unit, Karolinska Institute, Astrid Lindgren Children's Hospital Q6:05, S-171 76 Stockholm, Sweden

## Abstract

**Background:**

One of the most striking features of the childhood malignancy neuroblastoma (NB) is its clinical heterogeneity. Although there is a great need for better clinical and biological markers to distinguish between tumours with different severity and to improve treatment, no clear-cut prognostic factors have been found. Also, no major NB tumour suppressor genes have been identified.

**Methods:**

In this study we performed expression analysis by quantitative real-time PCR (QPCR) on primary NB tumours divided into two groups, of favourable and unfavourable outcome respectively. Candidate genes were selected on basis of lower expression in unfavourable tumour types compared to favourables in our microarray expression analysis. Selected genes were studied in two steps: (1) using TaqMan Low Density Arrays (TLDA) targeting 89 genes on a set of 12 NB tumour samples, and (2) 12 genes were selected from the TLDA analysis for verification using individual TaqMan assays in a new set of 13 NB tumour samples.

**Results:**

By TLDA analysis, 81 out of 87 genes were found to be significantly differentially expressed between groups, of which 14 have previously been reported as having an altered gene expression in NB. In the second verification round, seven out of 12 transcripts showed significantly lower expression in unfavourable NB tumours, *ATBF1*, *CACNA2D3*, *CNTNAP2*, *FUSIP1*, *GNB1*, *SLC35E2*, and *TFAP2B*. The gene that showed the highest fold change in the TLDA analysis, *POU4F2*, was investigated for epigenetic changes (CpG methylation) and mutations in order to explore the cause of the differential expression. Moreover, the fragile site gene *CNTNAP2 *that showed the largest fold change in verification group 2 was investigated for structural aberrations by copy number analysis. However, the analyses of *POU4F2 *and *CNTNAP2 *showed no genetic alterations that could explain a lower expression in unfavourable NB tumours.

**Conclusion:**

Through two steps of verification, seven transcripts were found to significantly discriminate between favourable and unfavourable NB tumours. Four of the transcripts, *CACNA2D3*, *GNB1*, *SLC35E2*, and *TFAP2B*, have been observed in previous microarray studies, and are in this study independently verified. Our results suggest these transcripts to be markers of malignancy, which could have a potential usefulness in the clinic.

## Background

Neuroblastoma (NB) is the most common extracranial solid tumour in children and accounts for around 15% of all childhood cancer deaths. It is a disease of the sympathetic nervous system and most often arises in the adrenal glands [1]. The most important prognostic factor in NB is clinical stage, which is based upon the local disease extension, degree of resection, and body dissemination [2,3]. Age at diagnosis is also an important factor, the younger the age the better the outcome, even when the disease is metastatic [4]. Although there is a number of commonly occurring genetic changes within the group of disease, no consensus alterations has been found that could explain the general pattern of tumourigenesis in these malignancies. Among the most frequent changes, which are also strongly associated with disease prognosis, are genomic amplification of MYCN (MNA), deletion of parts of chromosome arm 1p (del1p), partial deletion of 11q (del11q) and unbalanced gain of 17q. All these aberrations are correlated with poor outcome and clinical aggressiveness while whole chromosome gains or losses, and hyperdiploid/near-triploid cells define the more favourable tumour types [5]. Although the unbalanced chromosomal alterations affecting 1p, 11q and 17q have been known for some time, no single candidate gene has been unambiguously confirmed despite thorough mapping of these regions [6]. Recently, mutations of the Anaplastic Lymphoma Kinase (ALK) gene located on 2p23 ware found to be the main cause of familial NB [7]. ALK has also been found to be altered in sporadic NB tumours through either mutations (approximately 10%) or amplifications (approximately 5%) [7-11].

A particular hallmark of NB is its heterogeneity, in which some tumours regress spontaneously or with limited treatment while the most aggressive forms, especially in elder children (>1 year of age), have metastasised already at the time of diagnosis and are often resistant even to aggressive multimodal therapy [12]. These facts suggest divergent genetic mechanisms and pathways through which low- and high stage tumour types develop. Distinguishing tumour types by gene expression profiling has been a successful approach [13]. Moreover, expression analysis has already contributed to the finding of new markers and potential candidate genes that might be involved in tumour development [14-17]. Also, our group has identified an unbalanced expression of pro- and anti-apoptotic transcripts in unfavourable versus favourable tumours [16]. Since then, whole genome expression studies have shown differential expression patterns between different clinical stages [14] and between the biological groups of unfavourable and favourable tumours [15]. Wang and colleagues stated successful use of unsupervised hierarchical clustering in discriminating between tumours classified both according to stage and genetic alterations such as MNA and del1p. McArdle et al [18] also used hierarchical clustering and found 31 genes that could distinguish tumours on the basis of stage and differentiation. Moreover, an expression pattern highly correlated to deletion of chromosome 11q was observed. Gene expression can be affected by large structural genomic alterations, mutational events, or other inactivating mechanisms such as epigenetic alterations. Expression analysis of large genes located in genomic instability regions suggest that cancer progression is linked to inactivation of different fragile-site genes [19]. Also, distinct CpG island methylation patterns have been suggested to characterize different clinical groups of NB [20,21].

In the present study, we sought potential tumour suppressor genes by exploring gene expression differences between primary NB tumours with favourable or unfavourable biology from a Swedish patient group. The study is a verification of the results from a microarray analysis described previously [22]. In the current study, the selected transcripts from the microarray analysis were screened for differential expression by array-based quantitative PCR (QPCR). The data was further verified in a new set of tumours, and two candidate genes were analysed for inactivating genetic events.

## Methods

### Tumour samples

A set of 31 primary NB tumours of different stages was used in this study, 16 tumours were of favourable (F) biology and 15 tumours of unfavourable (UF) biology, see Table 1. Tumours were staged according to the International Neuroblastoma Staging System [2] and the International Neuroblastoma Risk Group Staging System (INRGSS) [23]. MNA and del1p status of tumour samples have been characterized in previous studies ([24,25] and unpublished data) using FISH analysis and microsatellite markers. Several of the tumours have also been investigated by Affymetrix 250K SNP array [26] to determine the MNA, del1p and del11q status (Table 1). Informed consent was obtained from the parents, and the study was approved by relevant ethics committees (Uppsala University d:nr 89/91, date: 15-05-1991). Tumour cases were assigned as favourable if staged 1–3 or 4S (according to INSS) with no MNA, del1p, or del11q, and no evidence of disease at last follow up. Tumour from a patient either dead of disease, with advanced stage of disease (stage 4) or with a stage 3 tumour (according to INSS) with either MNA, del1p, or del11q was classified as unfavourable. The treatment of the two cases with INSS stage 3 assigned as favourables (*i.e*. case 12E8 and 15E5) was as follows: case 12E8 was treated with non radical surgery only, and case 15E3 was treated with six courses of chemotherapy and uneventful radical surgery. Both patients show no evidence of disease, and have been followed up 17 years from diagnosis (until 1^st ^of January, 2009).

**Table 1:** Clinical data concerning primary neuroblastomas used in this study

**Group**	**Case**	**INSS **	**INRGSS**	**Age**	**Outcome**	**Ploidy**	**MNA**	**1p del**	**11q del**	**17q gain**	**OS**	**Ver. Group**	**Seq**	**CNA**
**F**	23R4	1	L								105	1	x	
	14E6^MA^	1	L								201	ni		
	
	18E8	1	L							WCG	169	1	x	
	31R8	1	L	**A**							73	1		
	35R8	1	L	I							50	1		
	26R9	1	L								91	2		
	16E1	1	L	I		**2n**					190	2		
	37R6	1	L								37	2		
	20R9	2	L			**2n**			nd	nd	113	1	x	
	33R7	2	L						nd	nd	62	1	x	
	25R9	2	L	**A**		**2n**					95	2		
	10R6	2	L			**2n**	nd	nd	nd	nd	160	2		
	13E8	2	L	**A**							202	2		
	
	12E8^MA^	3	L			**2n**				WCG	209	0		
	
	15E3^MA^	3	L				Gain	UB		WCG	195	0		
	21R6	4S	MS			**2n**					111	2		

**UF**	19R6	3	L		**DOD**	**2n**	**y**	**y**		**y**	8	1	x	
	
	16R4^MA^	3	L	**A**			**y**			**y**	131	0	x	
	
	9R9	3	M	**A**	**DOD**	**2n**		**y**	**y**	**y**	21	1	x	x
	10R8	3	L	**A**	**DOD**	**2n**		**y**	**y**	**y**	59	2		x
	
	10R2^MA^	4	M	I	**DOD**	**2n**	**y**	**y**		**y**	12	0		
	
	15R3^MA^	4	M	**A**	**DOD**	**2n**		**y**	nd	**y**	9	0	x	
	12E6	4	M	**A**	**DOD**	**2n**	**y**		nd	(y)	9	1		
	13R0	4	M	**A**	**DOD**	**2n**	**y**	**y**		**y**	10	1		
	
	26R8	4	M	I	**DOD**	**2n**	**y**	**y**	nd	nd	18	1		
	29R2	4	M	I		**2n**	**y**	**y**	nd	nd	85	1		
	
	11E1	4	M	**A**		**2n**			**y**	**y**	218	2		x
	26R0	4	M	**A**		**2n**	Gain	**y**	**y**	**y**	93	2		
	
	34R0	4	M	**A**	**DOD**	**2n**				**y**	11	2		x
	39R1	?	M	I		**2n**	**y**	**y**		**y**	29	2		x
	12E3	4	M	I	**DOD**	**2n**	**y**	**y**		**y**	4	2		x

Column 1: Group, F = Favourable, UF = unfavourable; column 2: Case ID, MA = Cases used in the Microarray study; column 3: Clinical stage according to International Neuroblastoma Staging System (INSS) [2]; column 4: Clinical stage according to International Neuroblastoma Risk Group Staging System (INRGSS) [23], L = locoregional tumors, M = metastatic tumors, MS = metastases are confined to the skin, liver, and/or bone marrow in children younger than 18 months of age.; column 5: Age = Age at diagnosis, B = Below 12 months, I = Intermediate, i.e. 12–24 months, A = Above 24 months; column 6: Outcome: DOD = Dead of disease, nd = not determined; column 7: Ploidy: 2n = Diploidy; column 8: MNA = MYCN amplification, y = MNA, gain = MYCN< 4 times the ploidy; column 9: 1p del = 1p deletion, y = 1p del, UB = unbalanced; column 10: 11q del = 11q deletion, y = 11q del; column 11: y = 17q gain, WCG = whole chromosome gain, (y) = uncertain results; column 12: Overall survival (OS) in months; column 13: Verification groups (Ver.Group) with QPCR, 0 = Replicate group, 1 = Verification group 1, 2 = Verification group 2, ni = not included; column 14: Seq = Cases used for DNA Sequencing or Bisulphite Sequencing PCR (BSP) of POU4F2; column 15: CNA = Cases used for Copy Number Aberration analysis of CNTNAP2.

### DNA/RNA extraction and cDNA synthesis

DNA was extracted using the DNeasy Blood- and Tissue kit (Qiagen, Hilden, Germany). RNA was extracted from collected tumour material after homogenisation by TissueLyser (Qiagen), using the Totally RNA kit (Ambion, St. Austin, TX). Genomic DNA was removed with the DNA-free kit (Ambion) and the purity and integrity of the RNA were assayed with the ND-1000 spectrophotometer (Saveen Werner AB, Malmö, Sweden) and RNA 6000 Nano Bioanalyzer (Agilent, Palo Alto, CA) respectively. Reverse transcription of total RNA was performed using High Capacity cDNA RT kit (Applied Biosystems, Foster City, CA), each reaction containing 1 μg RNA, 1× RT buffer, 4 mM dNTP mix, 1× random primers, 50 U reverse transcriptase and 20 U RNase inhibitor in a total volume of 20 μl. The reverse transcription reactions were run under the following conditions: 25°C for 10 min, 37°C for 120 min and 85°C for 5 seconds.

### Study design and selection criteria

**Figure 1:**
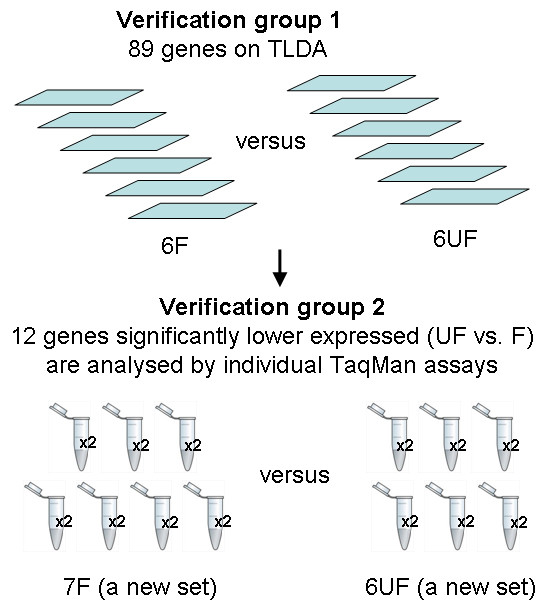
**Schematic representation of the study approach**. Verification group1: Eighty-nine genes were selected for gene expression analysis using TaqMan Low Density Array, TLDA, see text for details. In the TLDA analysis, six favourable (F) and six unfavourable (UF) tumours were included. Verification group 2: Twelve candidate genes were selected for validation by TaqMan individual QPCR, TM (see Table 3 for selection criteria). A new, randomly selected set of favourable (n = 7) and unfavourable (n= 6) tumours were used in the analyses, and all samples were run in duplicates (×2).

Twelve tumours, six favourable and six unfavourable tumours were used for the verification of our previous microarray data [22] by TaqMan Low Density Array (TLDA), see Verification Group 1 in Table 1. A new set of tumours, seven favourable and six unfavourable tumours were used for verification of TLDA data using individual TaqMan assays (Verification Group 2, Table 1). The selection of tumour samples for verification groups was random and no tumours were analysed in both verification experiments. The NB cell line SK-N-AS was used as a calibrator control to allow comparison of expression data between runs. The study design is illustrated in Figure 1.

The expression levels in five out of six tumours included in the microarray expression analysis (marked "MA" in Table 1) were confirmed by technical replication in the TLDA analysis of 89 transcripts. The material from the sixth tumour 14E6 was limited and was therefore excluded from the TLDA technical replicate study. The initial microarray data is publicly available on ArrayExpress , accession: E-MEXP-2250.

From the microarray study, transcripts were selected by the following criteria: i, probe sets with a fold change >2, ranked after significance level (p = 0,1E-05 to 0,05; calculated by ttest, [22]), ii, lower expression in unfavourable compared to favourable tumours. Transcripts represented by more than one probe set were included only when the majority of the probe sets showed a fold change >2. These stringent criteria resulted in a list of 88 transcripts. The *PHOX2B *gene was found on position 198 in the gene list, but due to its known involvement in NB it was also selected for the TLDA analysis. The first verification of 89 transcripts was analysed and the results guided the selection of twelve genes to be validated in verification group 2 (Table 1, Figure 1). These genes were selected only if significant (p < 0,05) and fulfilling one of two criteria: i, among the top ten genes with the highest fold change, ii, suggested to discriminate between groups in previous NB expression studies. In addition, one gene, *ATBF1*, was selected since it was the most significant and also previously reported to be involved in prostate and breast cancer. Another gene, *FUSIP1*, was selected due to its localization at chromosome arm 1p36. Four genes fulfilling criteria ii are parts of the noradrenic biosynthesis pathway and are described in detail elsewhere [22], and were not included in the second verification round.

### Real-time quantitative PCR (QPCR)

A total number of 17 NB tumours were investigated for 89 genes of interest and three endogenous controls, *i.e. ADA*, *GUSB *and *GAPDH*. The selection the endogenous controls is described in detail in Wilzén et al [22]. Custom-designed TLDA cards containing 95 individual assays were ordered from Applied Biosystems . Three samples and one calibrator (SK-N-AS) were loaded to each card according to manufacturer's instructions, each reservoir containing 83 ng of RNA converted to cDNA in a total volume of 100 μl. Twelve selected transcripts from the TLDA analysis were reanalysed with individual TaqMan assays in a new set of 12 tumours. The identical TaqMan assays as present on the TLDA cards were ordered separately from Applied Biosystems for the the second verification study. Individual QPCR reactions were set up in duplicates in 384 well plates using the Biomek FX pipetting robot (Beckman Coulter) and were carried out in 10 μl reactions with 1× TaqMan^® ^Gene Expression Mastermix (Applied Biosystems), 1× Gene-specific assay and 7,5 ng RNA converted into cDNA. Both TLDA cards and individual QPCR plates were run and analysed by the ABI PRISM^® ^7900HT Sequence Detection System (SDS 2.2, Applied Biosystems) according to manufacturer's protocol (Applied Biosystems).

### Expression data analysis

Calculations were performed using the ΔΔCt relative quantification method. The thresholds and baselines were set manually in SDS and Ct values were extracted. All Ct values were normalized to the mean of the endogenous controls *ADA*, *GUSB*, and *GAPDH *for each sample [22,27]. To evaluate the agreement between the microarray and QPCR expression levels in the technical replicate, a Pearson correlation coefficient was calculated for each gene.

Fold change between groups was calculated from the means of the logarithmic expression values. To be able to compare results between runs, all expression values were calibrated to the expression values of the NB cell line SK-N-AS, which was included in every real-time QPCR run. To confirm that the differences in expression were indeed representative for the two groups, a one-tailed, heteroscedastic student's t-test was performed for every transcript.

The final estimation of fold change and significance was based on the last verification group to avoid effect bias.

### Sequencing analysis

**Figure 2:**
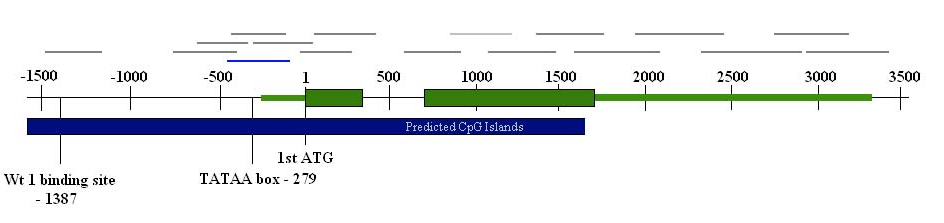
**Schematic representation of the *POU4F2 *gene**. Green lines represent exons where the green boxes specify the protein coding parts. Position 1 marks the translation start. The upper grey lines represent the fragments amplified by selected primer pairs. The lower dark blue box indicates predicted CpG islands  and the upper blue line marks the region covered by methylation analysis.

DNA sequencing analysis of the gene *POU4F2 *[GenBank: NM_004575] was performed on four favourable and four unfavourable primary tumours showing the lowest and highest expression values in their respective group (Table 1). Sixteen PCR-primer pairs covering the promoter region and the coding regions of *POU4F2 *were designed using Exon Primer  and purchased from Invitrogen (Invitrogen, Carlsbad, CA). Of these, four pairs span the 5'UTR/promoter region including one covering the Wilms' tumour transcription factor (Wt1) binding site located at -1387 to -1377 from translation start (Figure 2) [28]. Exon 1 was covered by three amplicons and exon 2 of nine amplicons (Figure 2). Primer sequences are available on request. Touch down (TD) PCR was performed in 10 μl reactions containing 1× Coral Load PCR Buffer (Qiagen), 20 mM dNTP mix, 1× Q-solution (Qiagen), 0,25 U Hot Star TaqPlus DNA polymerase (Qiagen), 10 μM of forward (FWD) and reverse (REV) primer, respectively, and 50 ng of tumour DNA. The TD PCR program was optimized for GC-rich fragments and run at 95°C for 15 min before cycling 20 rounds of 98°C for 30 sec, 60°C for 30 sec (decreasing 0,5°C in every cycle), and 72°C for one minute – followed by 25 cycles of 98°C for 30 sec, 50°C for 30 sec and 72°C for 1 min and finally a 72°C extension step for 7 minutes. Amplification products were analysed for specificity on a 2% agarose gel before they were purified using AMPure magnetic beads (Agencourt Bioscience Corporation, Beverly, MA) using the Biomek NX pipetting robot (Beckman Coulter) and eluted in dH_2_O. Sequencing PCR was performed using the BigDye Terminator (BDT) v3.1 Cycle Sequence Kit (Applied Biosystems) in 10 μl reactions containing 6 μl 1:3 diluted PCR-template DNA, 1 μl BDT, 1× BDT buffer and 1,6 μM of the PCR primer, either FWD or REV. Sequence PCR was run under following conditions; 94°C for 3 min, followed by 50 cycles of 96°C for 30 sec, 50°C for 10 sec and 60°C for 3 min each. Sequencing products were cleaned using CleanSeq magnetic beads (Agencourt) using the Biomek NX and resuspended in 10 μl of High Dye formamide (Applied Biosystems). The sequencing products were separated with gel electrophoresis on the 3730 DNA analyser (Applied Biosystems) and the output data were viewed and analysed using softwares Sequencing Analysis v 5.0 and SeqScape v 2.5, both from Applied Biosystems. All eight primary tumours and the positive reference CEPH DNA (CEPH1347-02, Applied Biosystems) were successfully sequenced. Each finding was validated by a second PCR and sequencing reaction.

### Bisulphite sequencing

Bisulphite sequencing PCR (BSP) was performed on the same eight primary tumours as described in the "Sequencing analysis" section (see above), as well as two NB cell lines SK-N-AS and SK-N-BE (Table 1). Bisulphite modification (BSM) of DNA was performed as described previously [21]. Prediction of the CpG islands within the *POU4F2 *promoter was performed with CpGproD and CpG island searcher  using the following criteria; an observed to expected CpG ratio over 0,65 and a GC content of over 55% in a stretch longer than 500 bp as described in Takai et al. [29]. Primers were designed using Bisearch [30]. Amplification of this region was performed with TD PCR (primer sequences available on request) according to the same procedures as previously described in the DNA amplification step of the sequencing experiment (see above). The amplification was repeated with an inner forward primer (semi-nested PCR) before purifying and sequencing of PCR products according to previously described procedures. Two amplicons of different length but starting with the same forward primer were sequenced. The shortest of the final amplification products covered 656 bp of the *POU4F2 *promoter (located at -655 to +1 from translation start, Figure 2).

### Copy Number Aberration (CNA) analysis

CNA analysis of the gene *CNTNAP2 *[GenBank: NM_014141] was performed using Single Nucleotide Polymorphism (SNP) microarrays from six primary tumours (see Table 1). The SNP microarray experiments have been described previously [26]. Briefly, Affymetrix 250 K SNP arrays (Nsp) were used and primary data analysis was performed using GDAS (GeneChip^® ^DNA Analysis software; Affymetrix), whereas further statistical studies were performed using CNAG (Copy Number Analyzer for Affymetrix GeneChip Mapping arrays software, version 3.0; Genome Laboratory, Tokyo University, ).

## Results

### QPCR expression analysis

#### Verification group 1

By TLDA analysis, the differential expressions of 81 out of 87 transcripts were significantly verified (Table 2). Two transcripts, *ITGAE *and *MGC12760*, showed undetectable expression values and were excluded from the study. A Pearson correlation calculation of expression values from the five technical replicates showed a good correlation between microarray and TLDA expression levels (average correlation coefficient = 0,7; see additional file 1). The transcript discriminating groups with the largest fold change in verification group 1 was *POU4F2 *(Table 2), showing an expression level of more than 1500 times lower (p = 0,011) in the unfavourable tumours compared to the favourable ones. Five tumours (4/5 unfavourable), did not express this gene at all (*i.e*. 25R9, 19R6, 9R9, 10R2, and 15R3). This absence of expression was verified in three rounds of individual QPCR runs, and the Ct-values were set to 40 in these cases to enable calculations.

**Table 2:** QPCR results of verification group 1

**Gene**	**Cytoband**	**t-test **	**Sign. **	**FC**	**Earlier suggested by**
ACD	16q22.1	0.1029	n.s.	4	
ACP1	2p25	0.0023	**	4	
**ATBF1**	**16q22.3**	**1.70E-05**	***	**9**	
BRSK2	11p15.5	2.40E-04	***	21	
C2orf25	2q23.3	0.0929	n.s.	3	
C5orf13	5q22.1	0.0102	*	4	
**CACNA2D3**	**3p21.1**	**0.0019**	**	**133**	[31]
CBFB	16q22.1	2.04E-05	***	9	
CCND1	11q13	0.0012	**	6	
CDC5L	6p21	0.0072	**	15	
CHCHD2	7p11.2	0.0041	**	4	
CLASP1	2q14.2-q14.3	0.0021	**	12	
**CNTNAP2**	**7q35-q36**	**0.0011**	**	**287**	
CXXC4	4q24	0.0350	*	10	
DBH	9q34	0.0039	**	15	[15,32]
**DCUN1D2**	**13q34**	**0.0027**	**	**26**	
DCX	Xq22.3-q23	0.0091	**	9	
DDC	7p11	0.0156	*	24	[32,33]
DGUOK	2p13	0.0047	**	10	
**DPYSL3**	**5q32**	**0.0100**	**	**23**	[33]
EIF2S3	Xp22.11	0.0024	**	4	
FLJ20323	7p21.3	0.0015	**	10	
FSCN1	7p22	0.0091	**	4	
FSD1	19p13.3	0.0077	**	8	
FUS	16p11.2	0.0350	*	15	
**FUSIP1**	**1p36.11**	**0.0128**	*	**22**	
GATA2	3q21.3	0.0260	*	4	[32]
GATA3	10p15	0.0018	**	6	[15]
GDF1; LASS1	19p12	0.0409	*	10	
**GNB1**	**1p36.33**	**0.0028**	**	**10**	[15,32]
H3F3B	1q41	0.0057	**	4	
HDAC2	6q21	0.0036	**	11	
HNRPDL	4q21.22	0.0025	**	7	
HNRPH3	10q22	0.0086	**	7	
IDH2	15q26.1	0.0023	**	6	
ILF2	1q21.3	0.0161	*	6	
ISL1	5q11.2	0.0074	**	6	
ITGAE	17p13	n.d.	n.d.	n.d.	
KIAA0408	6q22.33	3.96E-04	***	15	
KIDINS220	2p24	0.0014	**	15	
LOC440434	17q12	0.0264	*	11	
MAOA	Xp11.3	0.0105	*	12	
MARCKSL1	1p35.1	0.0107	*	5	
MCM6	2q21	0.0301	*	5	
MCG12760	1p36.13	n.d.	n.d.	n.d.	
MCG4655	16q22.1	0.3849	n.s.	1	
MTF2	1p22.1	7.83E-04	***	10	
NACA	12q13.3	0.0240	*	4	
NDUFS4	5q11.1	5.06E-04	***	5	
NONO	Xq13.1	0.0015	**	6	
NSUN6	10p12.31	0.0010	***	20	
PAFAH1B3	19q13.1	0.0044	**	8	
PALM	19p13.3	2.28E-04	***	7	
PHOX2A	11q13.2	0.0223	*	26	
PHOX2B	4p12	0.0042	**	18	[15]
PILRB	7q22.1	0.0084	**	15	
PKIA	8q21.11	8.69E-04	***	21	
POU2F1	1q24.1-24.2	5.77E-04	***	20	
**POU4F2**	**4q31.2**	**0.0106**	*	**1518**	[18,34]
PRKRA	2q31.2	0.0042	**	18	
RABL2B; RABL2A	2q13	0.0122	*	17	
**RAPGEF6**	**5q23.3**	**0.0097**	**	**37**	[35]
REV1L	2q11.2	8.99E-04	***	9	
RPL19	17q12	0.1034	n.s.	2	
SEC61G	7p11.2	0.0140	*	7	
SEPHS1	10p14	4.51E-04	***	6	
SFRS3	6p21	0.0126	*	5	
SHC1	1q21	0.0129	*	12	
SLC18A1	8p21.3	0.0130	*	25	
**SLC35E2**	**1p36.33**	**0.0241**	*	**3**	[15]
**SLC6A2**	**16q12.2**	**0.0012**	**	**540**	
SMN1; SMN2	5q13	0.0104	*	8	
SMPD4	2q21.1	0.0279	*	6	
SPAST	2p22.3	2.48E-05	***	9	
ST13	22q13.2	0.0324	*	5	
TAF9B	Xq21.1	0.0243	*	7	
TCP1	6q25.3	2.89E-04	***	6	
**TFAP2B**	**6p12.3**	**0.0115**	*	**13**	[14,31,33,34]
TH	11p15.5	0.0126	*	11	[15,32]
TIA1	2p14	5.99E-05	***	16	
TMSL8	Xq22.1	0.2105	n.s.	2	
TNFRSF25	1p36.31	0.0717	n.s.	8	[15,33,36]
TOP2B	3p24.2	2.49E-04	***	4	
TPRKB	2p13.2	0.0386	*	4	
UBE2E3	2q31.3	0.0110	*	13	
UCKL1	20q13.33	0.0013	**	11	
VPS28	8q24.3	0.0090	**	9	
XRCC5	2q35	0.0092	**	7	
YWHAQ	2p25.1	0.0400	*	5	

Ver 1 = verification group 1; Cytoband = Chromosomal location; t-test = significance by Student's t-test; n.d. = not determined; Sign. = Significance level by Student's t-test, * p < 0,05, ** p < 0,01, *** p < 0,001, n.s. = not significant; FC = Fold change between groups. Transcripts selected for the second verification are highlighted in bold.

#### Verification group 2

**Figure 3:**
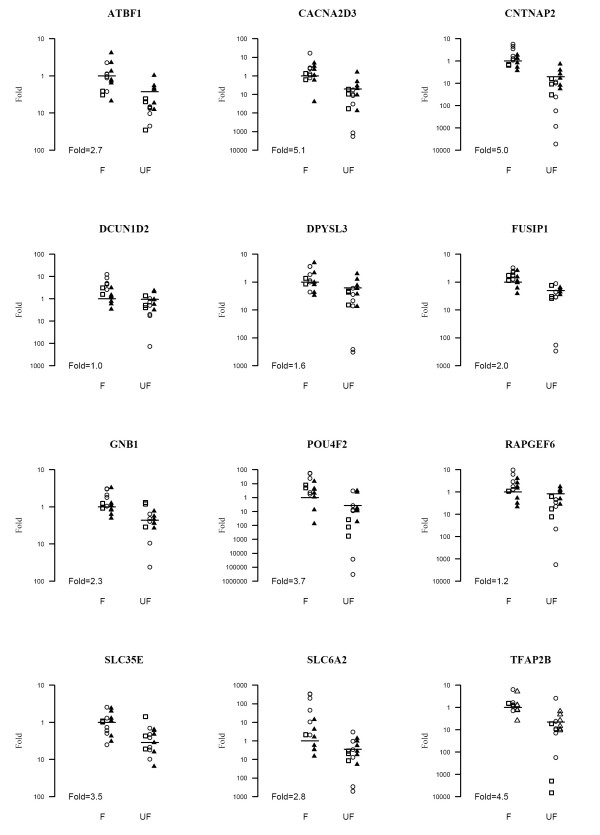
**Fold scatter plot of 12 transcripts studied by both TLDA and TaqMan**. The geometric means of the relative expression in favourable tumours of verification group 2 are used as reference (Fold = 1). Open squares = technical replicate group studied by TLDA but also represented on the microarray; Open circles = verification group 1 studied only with TLDA; Filled triangles = verification group 2 studied by individual TaqMan assays. The fold change (FC) between groups is based on expression values in verification group 2. Group: F = Favourable tumour types: Group UF = Unfavourable tumour types.

Twelve significantly differentially expressed transcripts from the TLDA study were selected for a second verification in 13 new tumour samples using individual TaqMan assays (with primers and probes targeting the same region as in the TLDA study). For seletion criteria and results see Table 3. Seven out of 12 genes could be significantly verified in this second round: *ATBF1*, *CACNA2D3*, *CNTNAP2*, *FUSIP1*, *GNB1*, *SLC35E2*, and *TFAP2B *(p < 0,05; Table 3 and Figure 3). The three transcripts, *CACNA2D3*, *CNTNAP2 *and *TFAP2B*, showed a 4–5 times lower expression in unfavourable tumour types (Figure 3).

**Table 3:** QPCR results of genes selected for the second verifiaction round

**Gene**	**Selection criteria**	**FC Ver 1**	**t-test Ver 1**	**FC Ver 2**	**t-test Ver 2**
ATBF1	p-value	9	1.70E-05	**2.7**	**0.032**
CACNA2D3	FC	133	0.0019	**5.1**	**0.049**
CNTNAP2	FC	287	0.0011	**5.1**	**0.0036**
DCUN1D2	FC	26	0.0027	**1.0**	**0.46**
DPYSL3	FC	23	0.01	**1.6**	**0.19**
FUSIP1	LOH	22	0.013	**2.0**	**0.013**
GNB1	PR	10	0.0028	**2.3**	**0.007**
POU4F2	PR; FC	1518	0.011	**3.7**	**0.15**
RAPGEF6	PR	37	0.0097	**1.2**	**0.36**
SLC35E2	PR	3	0.024	**3.5**	**0.011**
SLC6A2	FC	540	0.0012	**2.8**	**0.11**
TFAP2B	PR	13	0.012	**4.5**	**0.0049**

Column 2: p-value = Most significant in Ver 1 and cancer involvement, FC = Top ten with highest fold change (FC), LOH = Localized in a common Loss of Heterozygosity (LOH) region, PR = Previously Reported in NB microarray expression studies; column 3–4: Ver 1 = verification group 1; column 5–6: Ver 2 = verification group 2;

In order to test for potential influence of MNA, the 89 genes of the first verification round was compared to two curated MYC genesets, *i.e*. "MYC_ONCOGENIC_SIGNATURE" (212 genes) and "MYC_TARGETS" (42 genes), from Molecular Signatures Database at Broad Institute . None of the 89 genes overlapped with these two genesets. Moreover, a multiple linear regression study was performed on the 12 transcripts. The analysis showed that "group" (UF or F) remained the strongest predictor of differential expression compared to "MNA" (data not shown), which indicate that the altered expression levels of these transcripts are not a secondary effect of MNA.

### *POU4F2 *sequencing

To find out whether the dramatic expression variation of *POU4F2 *could be explained by differences at the genomic sequence we screened the promoter and coding parts of the gene for mutations by DNA sequencing in eight NB tumours (Table 1). The coding regions were void of mutations, neither did the Wt1 binding site nor the rest of the sequenced 5'UTR show any alterations compared the reference sequence. In two of the tumours, the 3'UTR also contained a known polymorphism, SNP rs 7669891, for which one of the tumours was homozygous and the other heterozygous (data not shown).

### *POU4F2 *methylation analysis

**Figure 4:**
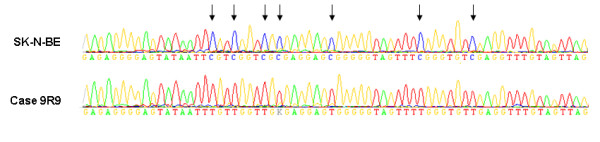
**BSP sequencing of the *POU4F2 *promoter**. Cell-line SK-N-BE shows methylation of CpG sites in the *POU4F2 *promoter. Cytosines (blue peaks) marked with arrows, are modified into thymidines (red peaks) if not methylated. Cell-line SK-N-BE is compared to unmethylated primary tumour 9R9.

Our other approach to find possible explanations for the differences in regulation of *POU4F2 *was to test whether the gene was epigenetically altered by promoter methylation. Preliminary results from methylation studies performed by our group indicate that the expression of *POU4F2 *increases in NB cell lines treated with a demethylating agent in conjunction with a histone deacetylase inhibitor (Carén et al, manuscript in preparation). DNA from the four unfavourables with the lowest expression and the four favourables with the highest expression of *POU4F2 *and two cell lines were tested for unmodified cytosines after bisulphite modification, which would indicate methylation of these loci. Although, one of the cell lines (SK-N-BE) was methylated at several CpG sites of the *POU4F2 *promoter, none of the primary tumours showed any remaining cytosines, and there were consequently no differences in promoter methylation between favourables and unfavourables (Figure 4).

### *CNTNAP2 *copy number analysis

In the current study, the mRNA level of *CNTNAP2 *was 5 times lower in unfavourables versus favourable tumours. This could indicate an inactivation by mutation or entire/partial deletion of the gene in unfavourable tumours. *CNTNAP2 *is one of the largest genes in the genome, and is located in a fragile site on 7q35-36. In order to find an explanation for the low expression in unfavourable neuroblastoma tumours, we scrutinized SNP microarray data from six unfavourable tumours (see Table 1) to search for deletions and other copy number aberrations of the *CNTNAP2 *gene (see additional file 2). Two tumour specimens (11E1, 9R9) showed gain of the whole chromosome 7q arm, but none of them showed deletions in the 7q35-36 region. Thus, the low expression of *CNTNAP2 *seen in these tumours does not seem to be caused by large genomic deletions, but an inactivation may still be caused by other mutational events.

## Discussion

Neuroblastoma is with its highly divergent clinical and genetic manifestations an elusive disease to study. Some characteristic genetic features have been found but none of them solely explains the variance in tumour behaviour and responsiveness to treatment. In the current study we performed a large-scale real-time PCR (TLDA) expression analysis to guide our selection of genes that are seemingly downregulated in unfavourable tumours, *i.e*. potential tumour suppressor genes. The natural approach would be to compare tumour tissue to its non-cancerous counterpart. However, since human normal embryonal sympathetic nervous system cells are not available for research studies, we chose to compare aggressive (unfavourable) to benign (favourable) tumours originating from the same type of precursor cells. A two step verification strategy was used, in which we started out by analysing the expression levels of 89 candidate transcripts, which were subsequently narrowed to 12 strong candidates for neuroblastoma progression. By TLDA analysis we were able to confirm the differential expression of 81 out of 87 transcripts seen in our microarray study. Fourteen of these transcripts have been suggested as differentially expressed in NB tumours in several independent research studies [14,15,18,31-36] (Table 2). To further strengthen our results we chose to perform a follow up study using a new set of tumours. In this second verification group we could confirm a lower gene expression in seven out of 12 genes (Table 3, Figure 3). Since the majority (9 out of 15) of unfavourable tumours show MNA, one could speculate that many of the differentially expressed transcripts could be part of the MYCN downstream signalling pathway and the lower expression would be merely an effect of MNA. However, a multiple linear regression of the second verification group showed that "group" (UF versus F) remained the strongest predictor of differential expression compared to "MNA", hence the downregulation of the seven transcripts seems independent to MNA. Moreover, the 89 genes selected for the first verification round are not included in MYC curated genesets from Molecular Signature Database (Broad Institute, ), seemingly the majority of differentially expressed transcripts verified in this study are not part of the MYCN downstream signalling network.

In this study the differential expression of *POU4F2 *was dramatic, as high as 1500 times difference in the first studied group of tumours. The large variance of expression was an effect of a complete depletion of the transcript in several unfavourable tumours in combination with a very high expression in some favourable tumours (Figure 3). POU4F2 has been shown to be essential for the development of retinal ganglion cells (RGC) [37] and has also been suggested to play an important role for a number of processes including proliferation and invasiveness [38,39], and development of different forms of malignancies. POU4F2 is a multi-functional protein shown to affect cell cycle protein [40,41], and to interact with several cancer-realted genes such as *BRCA1 *and *TP53 *[42,43]. Depletion of POU4F2 has been shown to make cells resistant to apoptosis, indicating a tumour suppressor function [43]. In a recent study, the *POU4F2 *transcript has actually, in contrary to our results, been found to be expressed at a higher level in primary tumours of higher stages, especially in stage 3 tumours correlating negatively with MNA [41]. On the other hand, the whole genome expression study of Wang and colleagues support our findings that *POU4F2 *show high expression in favourable tumours [15] and so does Albino et al. [34]. The *POU4F2 *or *BRN-3B *gene encodes a class IV POU (Pit-1, Oct1/2 and Unc-86) transcription factor mapped to 4q31.22. The *POU4F2 *gene is highly evolutionary conserved and show a high content of CpG sites indicating that the gene could be regulated by methylation (Figure 2). Moreover, the expression of *POU4F2 *gene is activated by the by the Wilms' tumour suppressor protein Wt1 [28] and has also been shown to have a highly conserved 3' UTR which through miRNA activity regulates the mRNA levels post-transcriptionally [39]. However, the mutation screeening of *POU4F2 *showed no apparent pathogenic alterations in the coding and regulatory parts of the gene, nor did we find any methylated CpG sites in the primary tumours, suggesting that the lower gene expression seen in UF tumours are not due to genetic alterations or DNA methylation. The only sample showing a few methylated CpG sites was the NB cell-line SK-N-BE (Figure 4). This result was in accordance to our previous observations where the expression levels of *POU4F2 *were up-regulated upon de-methylation of this cell-line.

Altogether, a differential expression of *POU4F2 *has been observed in several individual studies, which indicates an important function of this transcription factor during the process of NB tumour progression. Whether it functions as a survival factor or as a silenced tumour suppressor in these cases remain to be solved.

After the second verification round, *CNTNAP2 *showed the largest change in expression between groups of NB. *CNTNAP2*, or *CASPR2 *is one of the largest genes of the human genome, spanning over 2 Mb in size, encoding a protein localized in the complexes forming around specific K+ channels of myelinated axons [44]. Its location in the genome, 7q36, has been identified as a common fragile site (CFS), a large region more prone to show genomic instability with frequent deletions and other alterations [45]. The CFS genes have, both in *in vitro *and *in vivo *studies been suggested to be involved in development of cancer where they lose their function as tumour suppressor genes. McAvoy and colleagues found that *CNTNAP2 *was inactivated in brain tumours [19], which together with our findings support a tumour suppressor function of *CNTNAP2 *in neural cells. As *CNTNAP2 *gene is located in a CFS-site 7q35-36 it is not unlikely that the inactivation seen in unfavourable NB tumours is caused by entire or partial gene deletions at DNA level. However, copy number analysis of more than 300 SNP's covering *CNTNAP2 *showed that both alleles were retained in the genome in the six analysed tumours, and gene deletions are not likely to be the cause of the low expression seen in unfavourable tumours (see additional file 2). It is still possible that other genetic alterations may have affected the expression levels, and further studies of this gene are needed.

Also, *CACNA2D3 *and *TFAP2B *showed high differential expression between groups in the second verification round. *CACNA2D3 *encodes a calcium channel protein located on 3p22, a locus which is lost in more than half of the most aggressive NB cases especially in the absence of MNA [46]. A difference in expression between favourable and unfavourable NB has previously been observed by dePreter et al. [31]. Also, *CACNA2D3 *has been suggested to be a tumour suppressor gene in esophageal squamous cell carcinoma and has recently been found to be a poor prognostic factor in gastric cancer [47]. TFAP2B is one of the four members of the AP-class of transcription factors, plays a role in the retinoic acid-induced differentiation of neural crest cells, and is involved in the development of the kidney. Ebauer et al. has suggested that *TFAP2B *is the direct target gene of PAX3/FKHR fusion gene in alveolar rhabdomyosarcoma and responsible for the anti-apoptotic function of PAX3/FKHR [48]. Also, *TFAP2B *was recently suggested to be one of the genes discriminating between stroma-rich and stroma-poor neuroblastic tumours, in an approach similar to this study [34].

## Conclusion

In summary, several of the genes studied, particularly *CACNA2D3*, *CNTNAP2*, and *TFAP2B*, show a subgroup-specific expression pattern and could play a role in the development or maintenance of NB cancer cells. Apart from the noradrenergic pathway genes (*DBH*, *DCC*, *GATA2*, *GATA3*, *PHOX2A*, *PHOX2B*, *SLC6A2*, *SLC18A1*, and *TH*) [23], several previously reported genes have in this study been verified as differentially expressed genes, *i.e. CACNA2D3*, *DPYSL3*, *GNB1*, *POU4F2*, *RAPGEF6*, *SLC35E2*, and *TFAP2B *(Table 2 and 3) [14,15,18,32-37]. Down-regulation of these transcripts is a potential marker of tumour progression. Further routes of investigation are to analyse whether these changes in expression can be considered essential for the tumour progression and if these genes are deregulated because of alterations affecting them alone or if they are parts of affected pathways. Another issue is the possibility that the apparently down-regulated tumour suppressor genes in unfavourable tumours are in fact survival genes that are up-regulated in favourable ones, and further experimental studies are needed to confirm their tumour suppressor function.

In conclusion, this study verifies several differentially expressed transcripts that might have a potential clinical implication when it comes to increasing the accuracy of grouping and sub-grouping patients with NB, and guide for better treatment strategies. Further studies of these transcripts role in cellular networks will hopefully contribute to an even more detailed picture of the NB tumourigenesis and increase the understanding about this complex disease.

## Competing interests

The authors declare that they have no competing interests.

## Authors' contributions

KT carried out the real-time PCR and sequencing experiments, analyzed results and drafted the manuscript. FA formulated the study design, performed the microarray and TLDA analysis, supervised the calculations and interpretations of results, and revised the manuscript. AB contributed to the DNA copy number analysis and revised the manuscript. HC designed the methylation study and carried out the bisulphite modification. SN performed statistical analysis. TM provided DNA copy number data and performed the SNP microarray analysis, and provided clinical stages. PK provided clinical input on tumour classification and overall survival. All authors read and approved the final manuscript.

## Pre-publication history

The pre-publication history for this paper can be accessed here:



## Supplementary Material

Additional file 1 **Microarray versus QPCR: a correlation comparison of technical platforms**. Histogram over the Pearson correlation coefficients between microarray and QPCR expression levels of 87 genes analysed in 5 cases (technical replicates). The correlation calculation is based upon log2 expression values. Click here for file

Additional file 2 **Copy number analysis of *CNTNAP2***. 250 K SNP microarray data (Nsp) from six unfavourable NB tumours were scrutinized for entire or partial deletions in the 7q35-36 region. Click here for file
