# Supplementary figures and images for: Verification of genes differentially expressed in neuroblastoma tumours: a study of potential tumour suppressor genes
Source: BMC Med Genomics. 2009 Aug 17;2:53. doi: 10.1186/1755-8794-2-53 (PMC2743704; doi:10.1186/1755-8794-2-53)

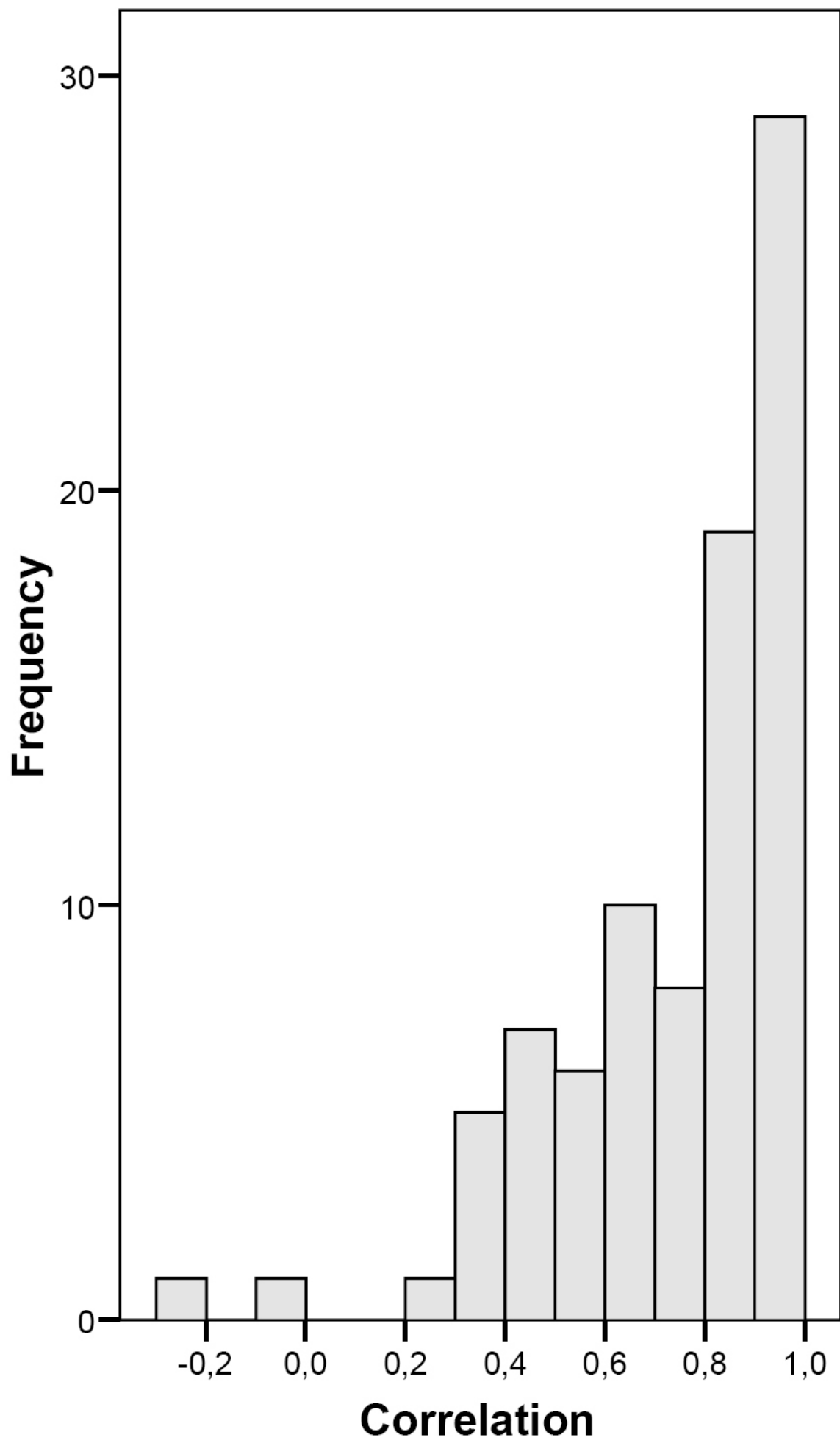

Supplement: Additional file 1 — Microarray versus QPCR: a correlation comparison of technical platforms. Histogram over the Pearson correlation coefficients between microarray and QPCR expression levels of 87 genes analysed in 5 cases (technical replicates). The correlation calculation is based upon log2 expression values. [file 1755-8794-2-53-S1.pdf]

Copy number analysis of chromosome 7

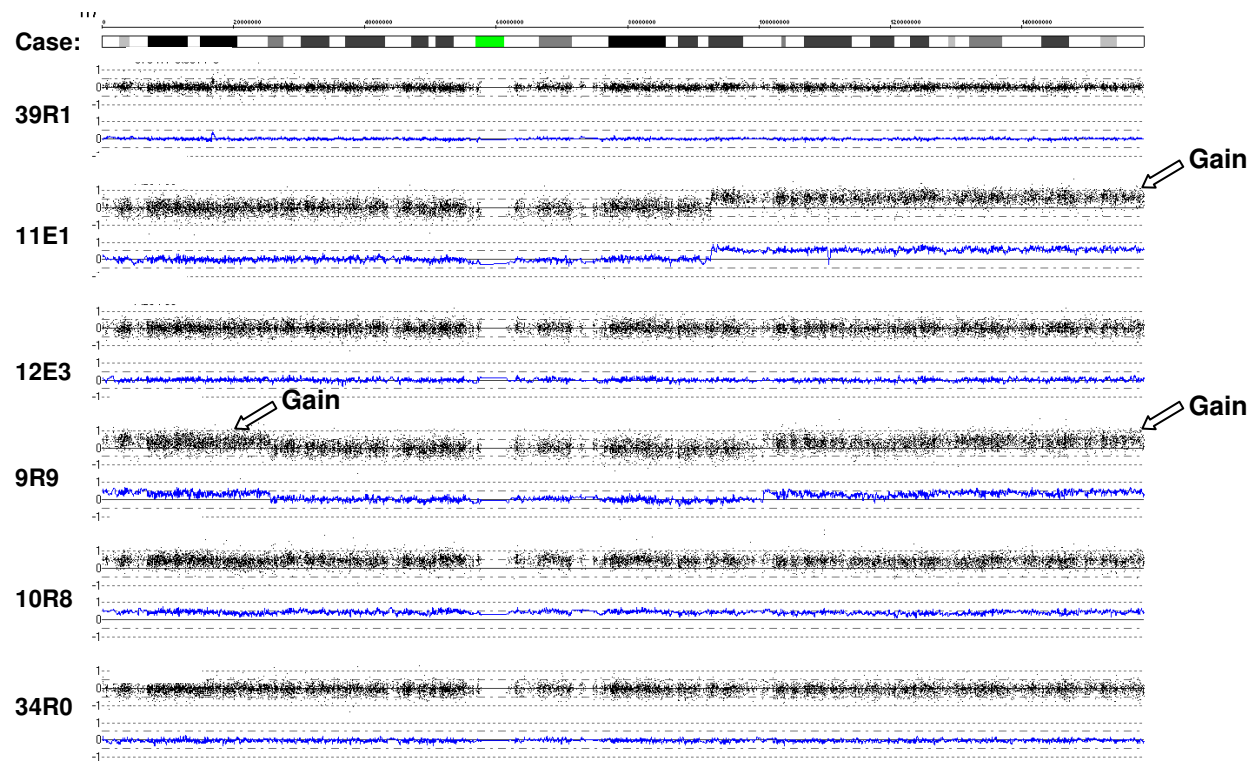

CNTNAP2 (www.genome.ucsc.edu)

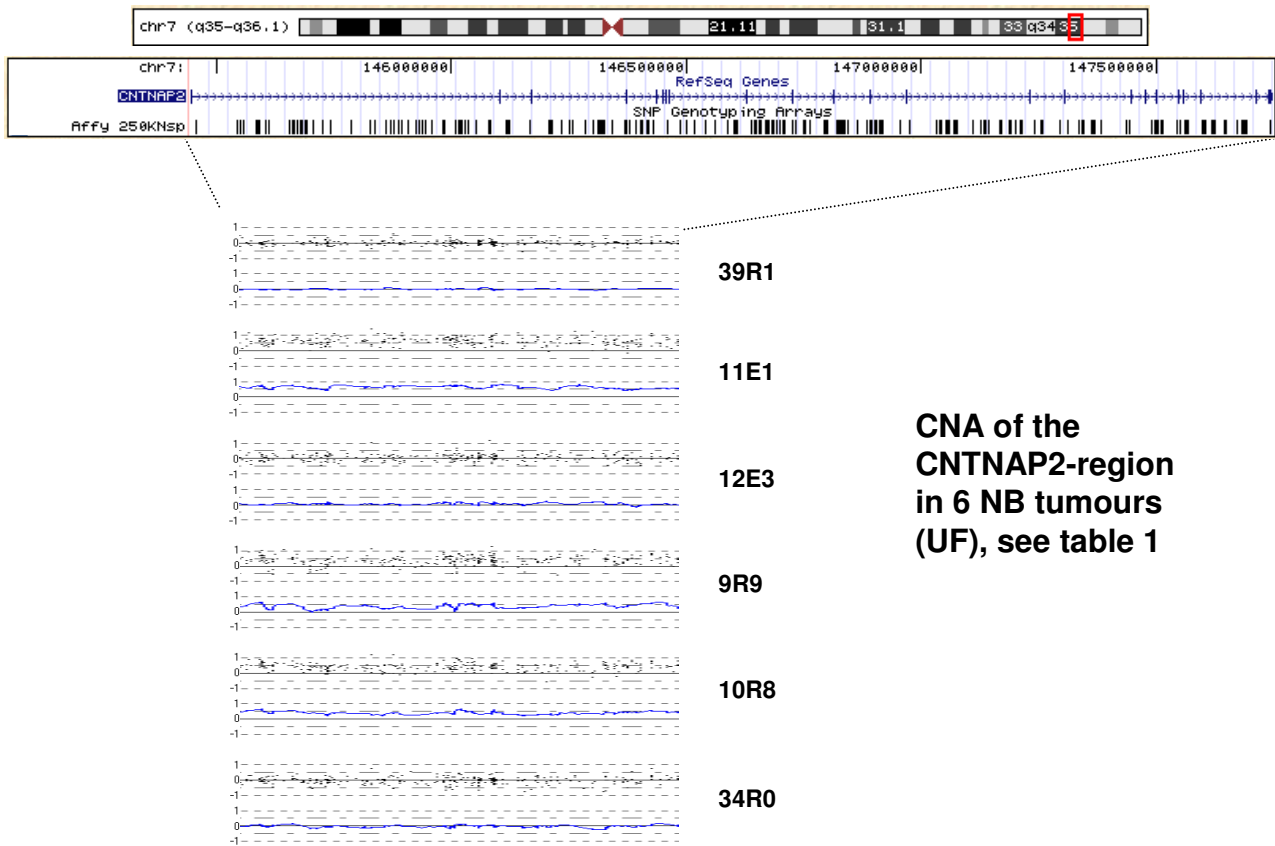

Supplement: Additional file 2 — Copy number analysis of CNTNAP2. 250 K SNP microarray data (Nsp) from six unfavourable NB tumours were scrutinized for entire or partial deletions in the 7q35-36 region. [file 1755-8794-2-53-S2.pdf]
